# Erratum to “The Challenge of Triaging Chest Pain Patients: The Bernese University Hospital Experience”

**DOI:** 10.1155/2012/493816

**Published:** 2012-01-02

**Authors:** Martin Rohacek, Amina Bertolotti, Nadine Grützmüller, Urs Simmen, Hans Marty, Heinz Zimmermann, Aristomenis Exadaktylos, Spyridon Arampatzis

**Affiliations:** ^1^Department of Emergency Medicine, University Hospital Bern, Freiburgstrasse, 3010 Bern, Switzerland; ^2^Statistical Consulting, Malzgase 9, 3042 Basel, Switzerland

In the original paper, the corresponding author's forename and surname were accidentally swapped. In this erratum, we state the correct version of the corresponding author's name, Spyridon Arampatzis, as requested.

